# An Important Role for CD4^+^ T Cells in Adaptive Immunity to Toxoplasma gondii in Mice Lacking the Transcription Factor Batf3

**DOI:** 10.1128/mSphere.00634-20

**Published:** 2020-07-15

**Authors:** Roxane Tussiwand, Michael S. Behnke, Nicole M. Kretzer, Gary E. Grajales-Reyes, Theresa L. Murphy, Robert D. Schreiber, Kenneth M. Murphy, L. David Sibley

**Affiliations:** a Department of Pathology and Immunology, Washington University in St. Louis School of Medicine, St. Louis, Missouri, USA; b Department of Molecular Microbiology, Washington University in St. Louis School of Medicine, St. Louis, Missouri, USA; c Howard Hughes Medical Institute, Washington University in St. Louis School of Medicine, St. Louis, Missouri, USA; University at Buffalo

**Keywords:** CD4 T cells, HIV-AIDS, chronic infection, dendritic cells, human immunodeficiency virus, opportunistic infection, toxoplasmosis

## Abstract

Immunity to Toxoplasma gondii at early stages of infection in C57BL/6 mice depends on gamma interferon (IFN-γ) production by NK cells, while at later stages it is primarily mediated by CD8 T cells. We decided to explore the requirement for CD4 T cells during T. gondii infection in *Batf3*^−/−^ mice, which lack CD8α^+^ dendritic cells (DCs) that are necessary for cross-presentation of cell-associated antigens to CD8 T cells. We show that in this immunodeficient background on a BALB/c background, CD4 T cells become important effector cells and are able to protect *Batf3*^−/−^ mice from infection with the avirulent strain RHΔ*ku80*Δ*rop5*. Independently of the initial NK cell activation, CD4 T cells in wild-type and *Batf3*^−/−^ mice were the major source of IFN-γ. Importantly, memory CD4 T cells were sufficient to provide protective immunity following transfer into *Batf3*^−/−^ mice and secondary challenge with the virulent RHΔ*ku80* strain. Collectively, these results show that under situations where CD8 cell responses are impaired, CD4 T cells provide an important alternative immune response to T. gondii.

**IMPORTANCE** Toxoplasma gondii is a widespread parasite of animals that causes zoonotic infections in humans. Although healthy individuals generally control the infection with only moderate symptoms, it causes serious illness in newborns and those with compromised immune systems such as HIV-infected AIDS patients. Because rodents are natural hosts for T. gondii, laboratory mice provide an excellent model for studying immune responses. Here, we used a combination of an attenuated mutant strain of the parasite that effectively vaccinates mice, with a defect in a transcriptional factor that impairs a critical subset of dendritic cells, to studying the immune response to infection. The findings reveal that in BALB/c mice, CD4 memory T cells play a dominant role in producing IFN-γ needed to control chronic infection. Hence, BALB/c mice may provide a more appropriate model for declining immunity seen in HIV-AIDS patients where loss of CD4 cells is associated with emergence of opportunistic infections.

## Abstract (precis)

Toxoplasma gondii is a widespread parasite of animals that causes zoonotic infections in humans. Although healthy individuals generally control the infection with only moderate symptoms, it causes serious illness in newborns and those with compromised immune systems such as HIV-infected AIDS patients. Because rodents are natural hosts for T. gondii, laboratory mice provide an excellent model for studying immune responses. Here, we used a combination of an attenuated mutant strain of the parasite that effectively vaccinates mice, with a defect in a transcriptional factor that impairs a critical subset of dendritic cells, to studying the immune response to infection. The findings reveal that in BALB/c mice, CD4 memory T cells play a dominant role in producing IFN-γ needed to control chronic infection. Hence, BALB/c mice may provide a more appropriate model for declining immunity seen in HIV-AIDS patients where loss of CD4 cells is associated with emergence of opportunistic infections.

## INTRODUCTION

Toxoplasma gondii is a promiscuous, obligate intracellular pathogen capable of infecting all types of nucleated cells from a wide range of warm-blooded animals (1). T. gondii replicates inside a protective parasitophorous vacuole, which segregates the parasite from the cytoplasmic environment and endosome/lysosome system of the host cell (2). Acute infection is established by rapidly replicating tachyzoites and is followed by chronic infection in which the parasite differentiates into bradyzoites that escape recognition and clearance by host immunity (3). During the acute phase of infection, the T. gondii protein profilin is recognized by the MyD88-dependent signaling pathway through recognition by TLR11 and TLR12 (4–6), which are expressed by CD8α^+^ conventional dendritic cells (cDCs) and tissue-resident CD103^+^ cDCs (7), as well as macrophages and epithelial cells (8). Early recognition of T. gondii or stimulation of CD8α^+^ DCs by soluble tachyzoite antigen (STAg) leads to the production of interleukin 12 (IL-12) (9, 10). *Batf3*^−/−^ mice, which lack CD8α^+^ and CD103^+^ cDCs, are therefore highly susceptible to infection with T. gondii due to inadequate early IL-12 production (11).

Immunity to T. gondii infection depends on IL-12 for the production of gamma interferon (IFN-γ) by NK cells early after infection, and by CD4 and CD8 T cells at later times (12, 13). Although early defense against T. gondii depends on CD8α^+^ cDCs (11), inflammatory monocytes and macrophages also produce IL-12, reinforcing the signal to produce IFN-γ (14, 15). Sustained levels of IFN-γ are necessary for control of acute and chronic infection, and the response to this cytokine is necessary on both hematopoietic cells and tissue cells (16–18). IFN-γ has numerous effects on cells, including the induction of immunity-related GTPases (IRGs), which are recruited to the parasitophorous vacuole and mediate its disruption (19). Recent evidence also implicates a second family of interferon-inducible GTPases, called the guanylate binding proteins (GBPs), which are also important in control of T. gondii infection (20). Host IRG and GBP proteins are counteracted by virulence factors expressed by type I strains of T. gondii, including the pseudokinase ROP5 and the active serine/threonine kinase ROP18, which together prevent recruitment of host effectors and rupture of the parasitophorous vacuole (21–25).

Adaptive immunity to T. gondii in C57BL/6 mice is primarily mediated by CD8 T cells, which are critical for controlling acute infection (26–28). In contrast to CD8 depletion, susceptibility of C57BL/6 mice is only marginally affected by CD4 T cell depletion, suggesting a dominant role for CD8 T cells in T. gondii immunity in the mouse (26, 27, 29). Furthermore, adoptive transfer of primed CD8 T cells, but not CD4 T cells, protects C57BL/6 mice against a secondary challenge with a lethal strain (30). CD4 T cells do play an important role during the priming phase of infection in C3H/HeN mice as their depletion during vaccination with avirulent strains of T. gondii prevents development of protective CD8 T cell immunity (31). CD4 T cells are likely important in part for their ability to produce IL-2 (26, 30, 32, 33). However, CD4 T cells are also an important alternative source of IFN-γ in C57BL/6 mice lacking both CD8 T cells and NK cells (34).

Here, we examined the effector function of CD4 T cells during T. gondii infection, using *Batf3*^−/−^ mice on a BALB/c background, in which NK and CD8 T cells are present and have normal intrinsic functions but lack CD8α^+^ cDCs needed for cross-presentation to CD8 T cells and for early IL-12 production (11, 35). We show that in the absence of CD8α^+^ cDCs, CD4 T cells become important effector cells in protective immunity to T. gondii infection in the mouse, highlighting a previously underappreciated role of CD4 T cells in the memory response.

## RESULTS

### Delayed activation of the innate response in *Batf3^−/−^* mice.

**FIG 1:**
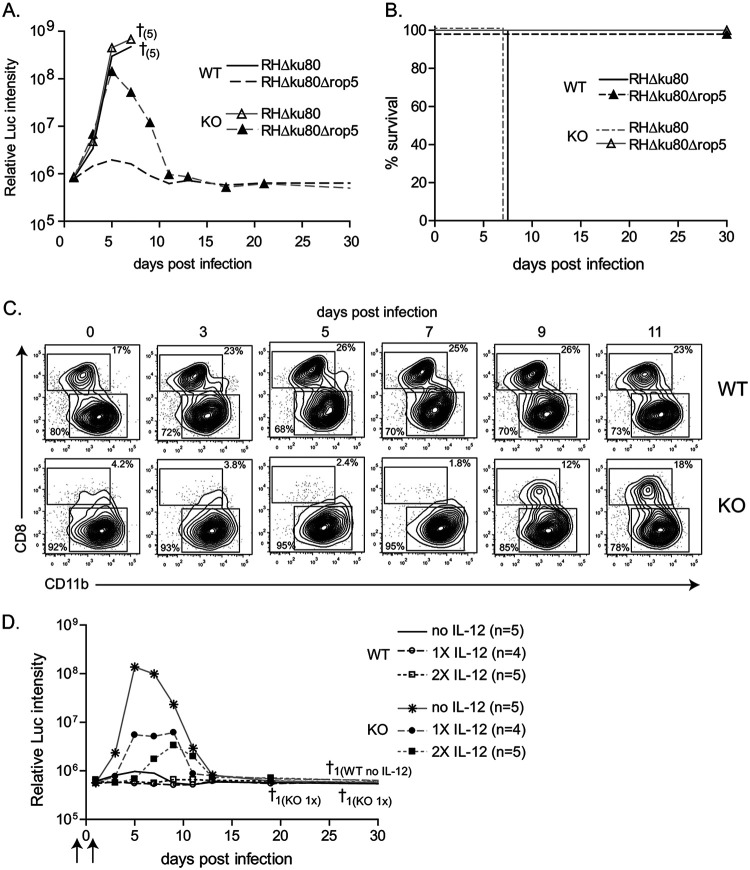
Delayed control of avirulent RHΔ*ku80*Δ*rop5* T. gondii infection in Batf3^−/−^ mice. (A and B) Wild-type (WT) and *Batf3^−/−^* (KO) mice were infected i.p. with 10^3^ RHΔ*ku80* or RHΔ*ku80*Δ*rop5* tachyzoites and monitored over 30 days for parasite growth (A) and survival (B). † indicates number of deaths (in parentheses) (*n* = 5). Results are representative of at least 2 independent experiments with similar outcomes. The background levels of luminescence in uninfected control mice are ∼10^6^. (C) Reappearance of CD8α^+^ DCs following infection with RHΔ*ku80*Δ*rop5* was monitored by flow cytometric analysis (FACS). Representative FACS plots of splenocytes from wild-type (WT) and *Batf3^−/−^* (KO) mice at the indicated time points pregated on CD11c^high^ MHC-II^high^ are shown. For each time point two mice were analyzed per group. The experiment was performed 2 times with similar outcomes. (D) Wild-type (WT) and *Batf3^−/−^* (KO) mice were left untreated (no IL-12), treated with recombinant IL-12 (arrows) 2 days before infection (1× IL-12), or 2 days before and 1 day after infection (2× IL-12) with 10^3^ RHΔ*ku80*Δ*rop5* tachyzoites and monitored over 30 days for parasite growth and survival. † indicates number of deaths and group; n indicates number of mice per group.

To examine the role of CD4 T cells in immunity to T. gondii infection, we used *Batf3*^−/−^ mice that lack CD8α^+^ and CD103^+^ cDCs in lymphoid and peripheral tissue, respectively (35). Since *Batf3*^−/−^ mice are highly susceptible to the type II Prugniaud (Pru) strain of T. gondii (11), which has intermediate virulence, we tested infection with the highly attenuated RHΔ*ku80*Δ*rop5* mutant (21, 22), which lacks the key virulence factor ROP5, a polymorphic serine threonine (S/T) protein kinase secreted from rhoptries (ROP) of T. gondii. As previously described (11, 21), infection of wild-type mice with the virulent parental RHΔ*ku80* strain resulted in a lethal outcome within 8 to 9 days (Fig. 1A and B). Similarly, *Batf3*^−/−^ mice also succumbed to infection with the RHΔ*ku80* strain, displaying comparable uncontrolled growth of the parasite (Fig. 1A and B). Wild-type mice infected with the attenuated RHΔ*ku80*Δ*rop5* parasite were able to effectively control infection (Fig. 1A). In contrast, *Batf3*^−/−^ mice infected with RHΔ*ku80*Δ*rop5* were initially unable to control growth but subsequently resolved and cleared the infection (Fig. 1A). The uncontained growth of the attenuated strain RHΔ*ku80*Δ*rop5* in Batf3^−/−^ mice at early time points is consistent with the known role of CD8α^+^ cDCs in controlling early T. gondii replication by driving IL-12 production. Nevertheless, *Batf3*^−/−^ mice uniformly survived infection by the RHΔ*ku80*Δ*rop5* strain (Fig. 1B), suggesting another mechanism was able to restore the ability to control infection.

To exclude the chance that pathogen clearance by *Batf3*^−/−^ mice was due to the previously described infection-mediated reappearance of CD8α^+^ cDCs (36), we analyzed the kinetics of CD8α^+^ DC reappearance following RHΔ*ku80*Δ*rop5* infection (Fig. 1C). CD8α^+^ cDCs were absent in *Batf3*^−/−^ mice before and up to 5 days after infection, reappearing only by day 9. However, *Batf3*^−/−^ mice began to clear infection by day 5, before the reappearance of CD8α^+^ cDCs (Fig. 1A), suggesting that clearance was not due to the reappearance of CD8α^+^ cDCs, but rather due to some other mechanism.

Because CD8α^+^ DCs mediate early secretion of IL-12 upon infection, we reasoned that adding exogenous IL-12 might compensate for their absence. Treatment with recombinant IL-12 was sufficient to reduce the magnitude and delay the initial burst of parasite growth (Fig. 1D). The parasite burden was controlled in a dose-dependent manner by exogenous IL-12 administration (Fig. 1D). A single dose of IL-12 was sufficient in wild-type mice to reduce parasite levels to the limit of detection. In *Batf3*^−/−^ mice a single dose of IL-12 reduced parasite levels significantly, and a second treatment 1 day after infection further delayed and limited the growth of RHΔ*ku80*Δ*rop5* (Fig. 1D).

**FIG 2:**
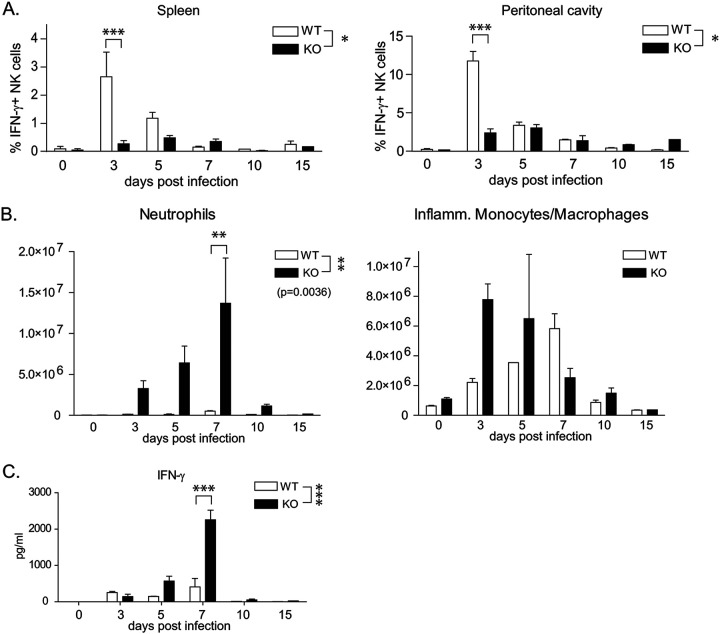
Contribution of innate immune subsets in the control of avirulent RHΔ*ku80*Δ*rop5* T. gondii infection. (A) Wild-type (WT) and *Batf3^−/−^* (KO) mice were infected (i.p.) with 10^3^ RHΔ*ku80*Δ*rop5* tachyzoites. The percentage of IFN-γ-positive NK cells in the spleen (left panel) and peritoneal cavity (right panel) of wild-type and *Batf3^−/−^* (KO) mice was analyzed by FACS over the course of infection. (*n* = 2 per time point). The experiment was performed 2 times with similar outcomes. Data are represented as means ± SD. (B) Wild-type (WT) and *Batf3^−/−^* (KO) mice were infected (i.p.) with 10^3^ RHΔ*ku80*Δ*rop5* tachyzoites. Indicated are the numbers of neutrophils (left panel) and inflammatory monocytes (right panel) recruited/present in the peritoneal cavity of wild-type and *Batf3^−/−^* mice. Counts were calculated based on the absolute numbers of cells collected from the peritoneal cavity and the percentage of cells obtained by FACS analysis (*n* = 2 per time point). The experiment was performed 2 times with similar outcomes. Data are represented as means ± SD. (C) Wild-type (WT) and *Batf3^−/−^* (KO) mice were infected i.p. with 10^3^ RHΔ*ku80*Δ*rop5* tachyzoites. Systemic IFN-γ levels were measured in the serum of infected mice (*n* = 2 per time point). The experiment was performed 2 times with similar outcomes. Data are represented as means ± SD. The statistical significance is indicated as follows: *, *P* ≤ 0.05; **, *P* ≤ 0.01; ***, *P* ≤ 0.001.

Early control of T. gondii infection involves IFN-γ production by NK cells. We therefore assessed NK cell activation by intracellular IFN-γ production in wild-type and *Batf3*^−/−^ mice infected with RHΔ*ku80*Δ*rop5* parasites (Fig. 2A; see also Fig. S1A in the supplemental material). IFN-γ production by NK cells in spleen and peritoneal cavity was detected in wild-type mice 3 days after infection and sustained until day 5 (Fig. 2A and Fig. S1A). In contrast, NK cell activation in *Batf3*^−/−^ mice was significantly reduced and delayed relative to wild-type mice (Fig. 2A and Fig. S1A), consistent with impaired recognition and IL-12 production in the absence of CD8α^+^ DCs (11).

10.1128/mSphere.00634-20.1 FIG S1 (A) Wild-type (WT) and *Batf3^−/−^* (KO) mice were infected (i.p.) with 10^3^ RHΔ*ku80*Δ*rop5* tachyzoites. The absolute number of IFN-γ-positive NK cells in the spleen (left panel) and peritoneal cavity (right panel) of wild-type and *Batf3^−/−^* mice was monitored over the course of infection. Counts were calculated based on the absolute numbers of cells collected and the percentage of cells obtained by FACS analysis (*n* = 2 per time point). The experiment was performed 2 times with similar outcomes. Data are represented as means ± SD. (B) Wild-type (WT) and *Batf3^−/−^* (KO) mice were infected with 10^3^ RHΔ*ku80*Δ*rop5* tachyzoites i.p. Systemic levels of the proinflammatory cytokines IL-6, MCP-1, and TNF-α were measured in the serum of infected mice (*n* = 2 per time point). The experiment was performed 2 times with similar outcomes. Data are represented as means ± SD. Download FIG S1, TIF file, 0.3 MB. Copyright © 2020 Tussiwand et al. 2020 Tussiwand et al. This content is distributed under the terms of the Creative Commons Attribution 4.0 International license.

Recruitment, differentiation, and maturation of inflammatory monocytes at the site of infection rely on NK cell activation and are critical for induction of sustained IL-12 production following intraperitoneal (i.p.) infection with T. gondii (37). Despite reduced activation and IFN-γ production by NK cells, *Batf3*^−/−^ mice showed greater recruitment of neutrophils and Ly6C^high^ inflammatory monocytes (Fig. 2B) to the site of infection than did wild-type mice. This difference may depend on the increased parasite burden in *Batf3*^−/−^ mice compared to wild-type mice, resulting in greater cell death and tissue damage. Consistent with this hypothesis, *Batf3*^−/−^ mice showed increased production of the inflammatory cytokines IL-6, CCL2, and tumor necrosis factor alpha (TNF-α) relative to wild-type mice (Fig. S1B). Increased inflammation may also explain the increased levels of systemic IFN-γ seen in Batf3^−/−^ mice after the parasite burden peaked (Fig. 1A and Fig. 2C). In summary, *Batf3*^−/−^ mice show increased parasite burden, inflammation, and systemic IFN-γ levels after infection by RHΔ*ku80*Δ*rop5* parasites relative to wild-type mice, but rather than succumbing to infection, they are able to clear the pathogen during the first weeks of infection.

### Clearance of the parasite occurs through CD4-specific IFN-γ production.

**FIG 3:**
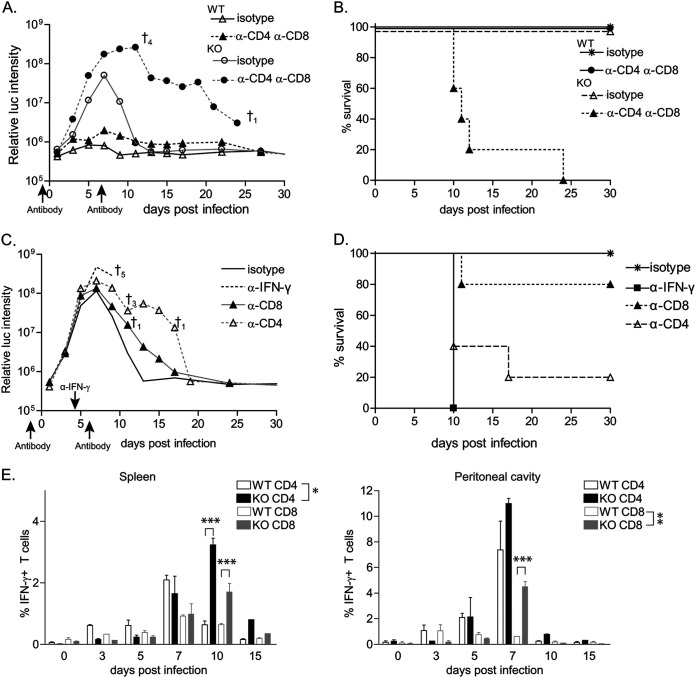
CD4 T cell-mediated clearance of infection via IFN-γ production. (A and B) *Batf3^−/−^* (KO) mice were injected with isotype or a combination of anti-CD4 and anti-CD8 depleting antibodies 1 day before and 7 days after infection with 10^3^ RHΔ*ku80*Δ*rop5* tachyzoites and monitored over 30 days for parasite growth (A) and survival (B). † indicates number of deaths (*n* = 5). (C and D) *Batf3^−/−^* (KO) mice were injected with isotype or depleting anti-CD4 or anti-CD8 or blocking anti-IFN-γ antibodies. Anti-CD4 and anti-CD8 antibodies were given 1 day before and 7 days after infection; anti-IFN-γ was given 4 days after infection with 10^3^ RHΔ*ku80*Δ*rop5* tachyzoites. Mice were monitored over 30 days for parasite growth (C) and survival (D). † indicates number of deaths (*n* = 5). (E) Wild-type (WT) and *Batf3^−/−^* (KO) mice were infected (i.p.) with 10^3^ RHΔ*ku80*Δ*rop5* tachyzoites. Percentages of IFN-γ-positive CD4 cells in the spleen (left panel) and peritoneal cavity (right panel) of wild-type and *Batf3^−/−^* mice were monitored over the course of infection. The experiment was performed 2 times with similar outcomes. Data are represented as means ± SD. The statistical significance is indicated as follows: *, *P* ≤ 0.05; **, *P* ≤ 0.01; ***, *P* ≤ 0.001.

The timing of parasite clearance around day 5, which coincided with delayed IFN-γ response, suggested that T cell-mediated immunity was involved in control of RHΔ*ku80*Δ*rop5* parasites. We therefore treated wild-type and *Batf3*^−/−^ mice with a combination of neutralizing anti-CD4 and anti-CD8 antibodies or isotype control antibody (Fig. 3A). *Batf3*^−/−^ mice succumbed to infection with RHΔ*ku80*Δ*rop5* parasites upon specific depletion of T cells, confirming that clearance relied on an efficient T cell response (Fig. 3A and B). In contrast, depletion of CD4 and CD8 T cells had no impact on RHΔ*ku80*Δ*rop5* parasite burdens in wild-type mice. These results indicate that infection with the highly attenuated RHΔ*ku80*Δ*rop5* strain can be eliminated independently of T cell function in wild-type mice, presumably due to early IFN-γ produced by NK cells (Fig. 2A).

Either CD8 or CD4 T cells or the combination of the two could be responsible for the observed clearance of RHΔ*ku80*Δ*rop5* parasites in *Batf3*^−/−^ mice. To test these alternative models, we depleted CD4 or CD8 T cells in *Batf3*^−/−^ mice and evaluated infection with RHΔ*ku80*Δ*rop5* parasites. Depletion of CD4 T cells caused lethality in the majority of mice, which succumbed by day 10 after infection (Fig. 3C and D). In contrast, single depletion of CD8 T cells had no effect on RHΔ*ku80*Δ*rop5* parasite burden in *Batf3*^−/−^ mice (Fig. 3C) and only a minor effect on overall survival of the mice (Fig. 3D). Pathogen clearance was dependent on IFN-γ, since administration of neutralizing antibody at the peak of infection on day 4 was uniformly lethal (Fig. 3C and D). Analysis of IFN-γ-secreting CD4 and CD8 T cells in the spleen and peritoneum revealed a different kinetic response between wild-type and *Batf3*^−/−^ mice (Fig. 3E and Fig. S2A). In wild-type mice, CD4 T cells began production 3 days after infection and peaked at day 7, when parasite burden had been reduced to the limit of detection. In *Batf3*^−/−^ mice, CD4 T cell activation was delayed until day 7 and peaked at day 10, after which parasites were eliminated (Fig. 3E and Fig. S2A). CD8 T cell responses followed a similar kinetic as CD4 T cell responses (Fig. 3E and Fig. S2A). Despite the absence of CD8α^+^ cDCs in *Batf3*^−/−^ mice, CD8 T cells produced significant amounts of IFN-γ at later time points and reached levels similar to wild-type mice, suggesting that the CD8 T cell response in *Batf3*^−/−^ mice was restored at later time points.

10.1128/mSphere.00634-20.2 FIG S2 (A) Wild-type (WT) and *Batf3^−/−^* (KO) mice were infected (i.p.) with 10^3^ RHΔ*ku80*Δ*rop5* tachyzoites. The absolute number of IFN-γ-positive CD4 and CD8 T cells in the spleen (left panel) and peritoneal cavity (right panel) of wild-type and *Batf3^−/−^* mice was monitored over the course of infection. Counts were calculated based on the absolute numbers of cells collected and the percentage of cells obtained by FACS analysis (*n* = 2 per time point). The experiment was performed 2 times with similar outcomes. Data are represented as means ± SD. Download FIG S2, TIF file, 0.2 MB. Copyright © 2020 Tussiwand et al. 2020 Tussiwand et al. This content is distributed under the terms of the Creative Commons Attribution 4.0 International license.

### CD4 T cells are sufficient to protect *Batf3^−/−^* mice in an adoptive transfer model.

**FIG 4:**
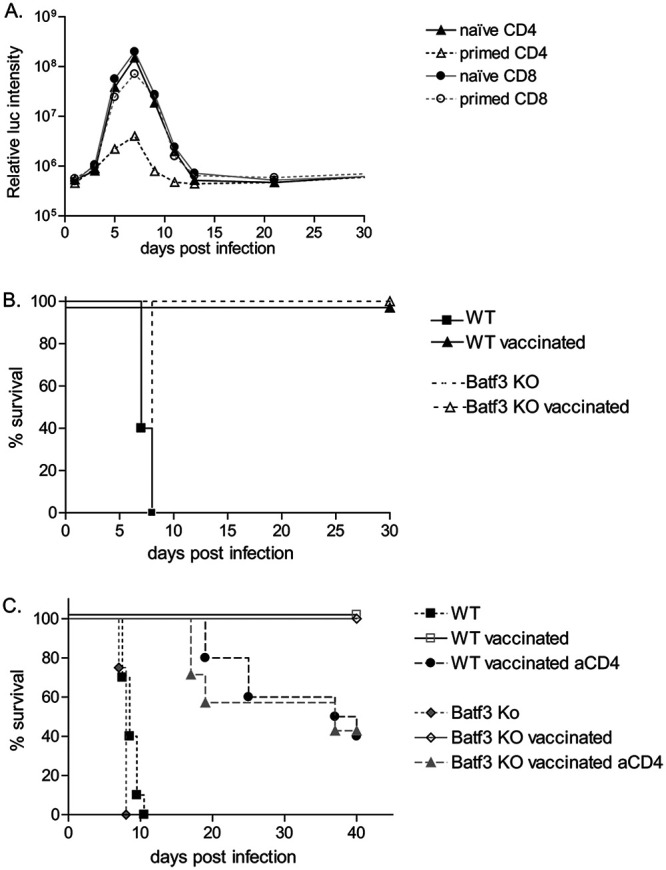
Primed CD4 T cells are sufficient to control parasite burden in Batf3^−/−^ mice. (A) Primed T cells were harvested from the spleen of *Batf3^−/−^* (KO) mice 21 days postinfection. Naïve or primed T cells (as indicated) were transferred into *Batf3^−/−^* (KO) mice 2 days before infection with 10^3^ RHΔ*ku80*Δ*rop5* tachyzoites. Mice were monitored over 30 days for parasite growth and survival (*n* = 4). (B) Wild-type (WT) and *Batf3^−/−^* (KO) mice were vaccinated with 10^3^ RHΔ*ku80*Δ*rop5* tachyzoites or medium alone as indicated and challenged 30 days later with 10^3^ RHΔ*ku80* tachyzoites. Survival after challenge was monitored (*n* = 5). (C) Wild-type (WT) and *Batf3^−/−^* (KO) mice were untreated or vaccinated with 10^3^ RHΔ*ku80*Δ*rop5* tachyzoites. Thirty days later, vaccinated wild-type and *Batf3^−/−^* (KO) mice were injected with isotype control or anti-CD4 depleting antibody, before challenge with 10^3^ RHΔ*ku80* tachyzoites. Survival after challenge was monitored (*n* = 10 except for WT and KO untreated [*n* = 9]; results combined from 2 independent experiments).

To further confirm that CD4 T cells were the major effectors of parasite clearance, we performed adoptive transfer experiments. *Batf3*^−/−^ donor mice were infected with RHΔ*ku80*Δ*rop5* parasites. After clearance of the parasites and contraction of the T cell population 30 days postinfection, CD4 and CD8 T cells were collected from spleens and transferred into nonirradiated *Batf3*^−/−^ recipients. *Batf3*^−/−^ naive recipient mice received either CD4 or CD8 T cells from *Batf3*^−/−^ naive or infected mice 1 day before infection with RHΔ*ku80*Δ*rop5* parasites and were monitored for 30 days. As expected, transfer of naive CD4 or CD8 T cells did not affect the parasite burden (Fig. 4A). Similarly, primed CD8 T cell transfer was not protective, with mice displaying a comparable parasite load as following transfer with naive T cells. However, primed CD4 T cells were sufficient to reduce the growth of RHΔ*ku80*Δ*rop5* parasites in *Batf3*^−/−^ mice, showing a similar parasite burden as RHΔ*ku80*Δ*rop5*-infected wild-type mice (Fig. 1A and Fig. 4A). These studies demonstrate the effector potential of primed CD4 T cells.

### CD4 memory T cells protect *Batf3^−/−^* and WT mice in a vaccination model.

Infection with RHΔ*ku80*Δ*rop5* was previously shown to protect mice from secondary challenge with the parental RHΔ*ku80* strain, which is normally uniformly lethal (21). Previous experiments with type I strains of T. gondii showed that the establishment of protective immunity by vaccination with avirulent strains mostly depends on CD8 T cells (30). Given the importance of CD4 T cell responses in *Batf3*^−/−^ mice in limiting RHΔ*ku80*Δ*rop5* parasite burdens (Fig. 3E, Fig. 4A, and Fig. S2A), we decided to examine the contribution of CD8 and CD4 T cells in protective immunity. Wild-type and *Batf3*^−/−^ mice were infected with avirulent RHΔ*ku80*Δ*rop5* parasites, and after 30 days they were challenged with virulent RHΔ*ku80* parasites and monitored for 30 days. Wild-type as well as *Batf3*^−/−^ mice survived the secondary challenge with wild-type parasites (Fig. 4B), suggesting that adaptive responses to primary infection were sufficient to induce long-term memory response. Since CD4 T cells were previously shown to be required during the priming phase for the establishment of an effective CD8 T cell response but not as effector cells, we performed CD4 depletion before the challenge phase with the virulent strain. Consistent with the results obtained for *Batf3*^−/−^ infection with the avirulent strain RHΔ*ku80*Δ*rop5* (Fig. 4A), depletion of CD4 T cells before challenge with RHΔ*ku80* significantly affected survival of *Batf3*^−/−^ mice (Fig. 4C). Increased susceptibility was also observed in wild-type mice (Fig. 4C), suggesting that CD4 T cells contribute not only in providing help to CD8 T cells during the priming phase but also as effector cells in controlling and clearing the parasite.

## DISCUSSION

Previous studies have shown that *Batf3*^−/−^ mice, which lack CD8α^+^ cDCs, are unable to control infection following challenge with strains of T. gondii that have intermediate virulence due to defective recognition of the parasite at early time points after infection, resulting in reduced IL-12 production and hence defective NK cell activation (11, 36) However, in Batf3^−/−^ mice, CD8 T cell priming can still occur through presentation of soluble antigens (38), as well as direct presentation, suggesting that adaptive immunity may only be partially affected. Here, we examined the ability of Batf3^−/−^ mice to control infection with a highly attenuated parasite mutant to distinguish between a requirement during innate (IL-12 production) and that during adaptive (cross-presentation to CD8 T cells) immunity. In this context, we show that CD4 T cell production of IFN-γ becomes critical to control of infection. Selective depletion or adoptive transfer of T cell subsets revealed that CD4 cells contribute more to production of IFN-γ and to protective responses than do CD8 T cells. Collectively, our results highlight a previously underappreciated role for CD4 T cells in mediating protective immunity, a finding that may have consequences for immunocompromised patients infected with T. gondii.

In wild-type mice, the initial recognition of T. gondii by TLR11 and TLR12 expressed by CD8α^+^ cDCs triggers early IL-12 production and NK cell-mediated secretion of IFN-γ leading to control parasite infection (4, 6, 39). In *Batf3*^−/−^ mice, this early IL-12 production is absent (11) with an increased parasite burden leading to greater proinflammatory cell recruitment. Despite the increased recruitment of inflammatory monocytes to the peritoneal cavity of Batf3^−/−^ mice, this did not lead to parasite control. This failure to control the parasite numbers may be a consequence of low levels of IFN-γ produced by resident NK cells and delayed levels of production by CD4 T cells in Batf3^−/−^ mice. As such, even though inflammatory monocytes are recruited to the site of infection, their lower capacity for IL-12 production and consequent lower levels of IFN-γ at these early time points impair the development of antimicrobial functions. Furthermore, our findings indicate that control of attenuated strains of T. gondii in Batf3^−/−^ mice is dependent on the function of CD4 T cells, as shown by depletion studies, rather than simply a reappearance of CD8α cDCs, which are eventually rescued though production of IL-12 (36). Previous studies have shown that this restoration of CD8α cDCs is dependent on compensatory function of other Batf transcription factors that interact with Irf4 and Irf8 to activate gene expression (36). However, these prior studies did not examine the role of CD4 T cells in control of chronic infection, as they were focused exclusively on the innate phase of immunity. Our current studies reveal that despite this return of CD8α DCs, CD4 T cells remain the dominant subset contributing to IFN-γ levels needed to control toxoplasmosis.

Although protection against toxoplasmosis in the immunocompetent C57BL/c mice is largely dependent on CD8 T cells, in the absence of NK cells and CD8 T cells, CD4 T cells become an essential source for IFN-γ (34). Similarly, because *Batf3*^−/−^ mice have impaired cross-presentation of cell-associated but not soluble antigens to CD8 T cells (38), we reasoned that adaptive immunity in these mice might be more dependent on CD4 T cell function. Consistent with this prediction, we show that in *Batf3*^−/−^ mice on a BALB/c background, CD4 T cells contribute to control of parasite infection. Selective depletion of CD4, but not CD8, T cells led to increased mortality in chronically infected Batf3^−/−^ and wild-type BALB/c mice. The greater role for CD4 T cells in this model may be due to their higher production of IFN-γ. The finding that CD4 cells play a more important role in the present study differs from previous studies using vaccination with a strain of intermediate virulence (type II ME49 strain) where CD8 cells were predominant (26, 30, 32). These differences may result from unique mechanisms of activation caused by the attenuated RHΔ*ku80*Δ*rop5* strain or reflect differences in the use of BALB/c mice here from previous studies that focused on C57BL/6 mice. Regardless, neutralization of IFN-γ was detrimental and resulted in the death of all RHΔ*ku80*Δ*rop5*-vaccinated animals, indicating that the likely mechanism by which CD4 T cells induce parasite clearance is through secretion of IFN-γ. This observation highlights the critical role of IFN-γ-induced pathways in promoting lysis of the parasitophorous vacuole, a process that occurs through recruitment of IRGs (19) and GBPs (40–42), thereby limiting and controlling the infection. Collectively, these studies highlight the remarkable flexibility of the immune system, which provides layers of redundancy needed to respond to pathogens under a variety of circumstances.

CD4 T cells normally play a minor role in control of T. gondii infection in immune sufficient hosts, where infections are usually well contained and largely asymptomatic. Consistent with this, in wild-type mice with the avirulent RHΔ*ku80*Δ*rop5* strain, depletion of CD4 T cells did not affect the parasite burden or survival. Innate protection mediated by activated NK cells, and production of IFN-γ, is apparently sufficient for clearance of this highly attenuated strain. However, in both *Batf3*^−/−^ and wild-type mice, CD4 memory T cells contributed to the protection of mice after challenge with lethal infection. This unanticipated result indicates that CD4 T cells contribute to immunity against T. gondii infection in chronically infected animals, as shown previously using C3H/HeN mice (31). This finding is also highly relevant to situations where the parasite burden may increase due to reactivation. In particular, T. gondii reactivation during immune suppression or in AIDS patients leads to severe and widespread tissue damage with a potential lethal outcome if left untreated (43). During the progression to AIDS, T. gondii reactivation results in encephalitis, and the progressive loss of CD4 T cells is associated with the progression of toxoplasmosis, supporting the relevance of CD4 T cell-mediated immunity. Hence, findings from BALB/c mice may provide an improved model to study the pathology of opportunistic infections in immunocompromised patients such as those with HIV-AIDS.

## MATERIALS AND METHODS

### Mice.

Wild-type BALB/c mice were originally purchased from Taconic and then bred in-house for experimental use. *Batf3^−/−^* mice were previously generated in our laboratory on a 129S6/SvEv background (35) and subsequently backcrossed for 10 generations onto BALB/c backgrounds. Mice were age and sex matched for each experiment and were between 8 and 12 weeks old. All mice were maintained under specific-pathogen-free conditions according to institutional guidelines and with protocols approved by the Animal Studies Committee of Washington University.

### Parasites and infections.

Luciferase-expressing parasite strains for type I virulent RH (RH*Δku80*) and an avirulent RH mutant (RH*Δku80Δrop5*) were grown in culture in human foreskin fibroblasts as previously described (21). For infections, parasites were harvested and counted, and 1,000 tachyzoites were injected i.p. into mice. Parasite burdens were measured by bioluminescence using luciferase-expressing parasites, as previously described (21). For survival experiments, mice were monitored daily over 30 days.

### Luciferase imaging.

Mice were injected i.p. with d-luciferin (Biosynth AG) at 150 mg/kg of body weight, anesthetized with 2% isoflurane for 5 min, and imaged with a Xenogen IVIS 200 imager, and images were processed using Xenogen Living Image software (Caliper Life Sciences).

### Cell preparation.

Spleens were digested in 5 ml complete Iscove’s modified Dulbecco’s medium (IMDM) with 250 μg/ml collagenase B (Roche) and 30 U/ml DNase I (Sigma-Aldrich) for 30 min at 37°C with agitation using stir bars. Red blood cells were lysed by incubation in ACK (ammonium-chloride-potassium) lysis buffer. Cells were filtered through 80-μm strainers and counted on an analyzer (Vi-CELL; Beckman Coulter). Cells (1 × 10^6^ to 5 × 10^6^) were stained for flow cytometric analysis. For analysis of peritoneal cells, a peritoneal lavage was performed with 10 ml Dulbecco’s phosphate-buffered saline (DPBS). Harvested cells were lysed in ACK buffer, filtered, counted, and stained for flow cytometry.

### Flow cytometry and staining.

Cells were incubated for 5 min at 4°C with Fc Block (clone 2.4G2; BD) in FACS buffer (PBS, 0.5% bovine serum albumin [BSA], 2 mM EDTA). Dead cells were excluded using the LIVE/DEAD Aqua fixable dead-cell stain kit (Invitrogen). Surface staining was done for 20 min at 4°C in FACS buffer. Absolute cell numbers were calculated using the total cell count multiplied successively by the percentages for the appropriate gates obtained through flow cytometry. Cells were analyzed on a BD FACS Canto II flow cytometer, and data were analyzed using FlowJo software (Tree Star, Inc.). Immune subsets were identified as previously described (11). Cell types were defined by the following markers: neutrophils, Ly6^hi^, F4/80^−^, Ly6C^lo^, CD11b^+^; inflammatory macrophages/monocytes, Ly6G^−^, Ly6C^hi^, CD115^hi^, CD11c^−^; resident macrophages, Ly6G^−^, F4/80^+^, CD115^hi^, CD11c^−^; CD8α dendritic cells, CD11c^+^, MHC-II^hi^, DEC205^+^, CD103^+^, CD8a^+^, CD11b^−^, Sirpα^−^; CD4^+^ dendritic cells, CD11c^+^, MHC-II^hi^, CD11b^+^, Sirpα^+^.

### Intracellular cytokine staining.

For intracellular cytokine staining, cells were first surface stained and then fixed in 2% paraformaldehyde for 15 min at room temperature. Cells were then resuspended in permeabilization buffer (PBS + 0.1% BSA + 0.5% saponin) and stained with anti-IFN-γ for 30 min at 4°C.

### Cytokine measurement.

Serum cytokine levels were measured using the BD CBA mouse inflammation kit (BD Biosciences). Detection was performed using a FACS Canto II flow cytometer (BD Biosciences), and results were analyzed using FCAP Array (Soft Flow, Inc.).

### Administration of IL-12.

Recombinant murine IL-12 (Peprotech) was resuspended in pyrogen-free saline at a concentration of 2.5 μg/ml, aliquoted, and frozen at −80°C. Mice were injected i.p. with 0.5 μg of IL-12 as indicated.

### IFN-γ and T cell depletion.

Mice were injected i.p. with 250 μg of anti-CD4 (clone GK1.5) and/or CD8 (clone H35) 2 days before infection, with a second dose given at day 5 postinfection. Depletion was monitored on peripheral blood. Mice were injected i.p. with 250 μg of IFN-γ-blocking antibody H22, or control antibody PIP, as previously described (17).

### T cell adoptive transfer.

CD4 (clone L3T4) and CD8 (clone Ly2) positive T cells were obtained by positive selection using microbead-based magnetically activated cell sorting (MACS) purification (Miltenyi Biotec). Purity was confirmed by fluorescence-activated cell sorting (FACS) analysis above 93%, and cells were injected intravenously 2 days before infection.

### Statistics.

For analyses of survival data, the log rank test was used. For analyses of all other data, a two-way analysis of variance (ANOVA) was used with posttest correction using the Bonferroni method (Prism; GraphPad Software, Inc.). All data are represented as means ± SD. The statistical significance is indicated as follows: *, *P* < 0.05; **, *P* < 0.01; ***, *P* < 0.001.
