# Supplementary figures and images for: An Important Role for CD4+ T Cells in Adaptive Immunity to Toxoplasma gondii in Mice Lacking the Transcription Factor Batf3
Source: mSphere. 2020 Jul 15;5(4):e00634-20. doi: 10.1128/mSphere.00634-20 (PMC7364223; doi:10.1128/mSphere.00634-20)

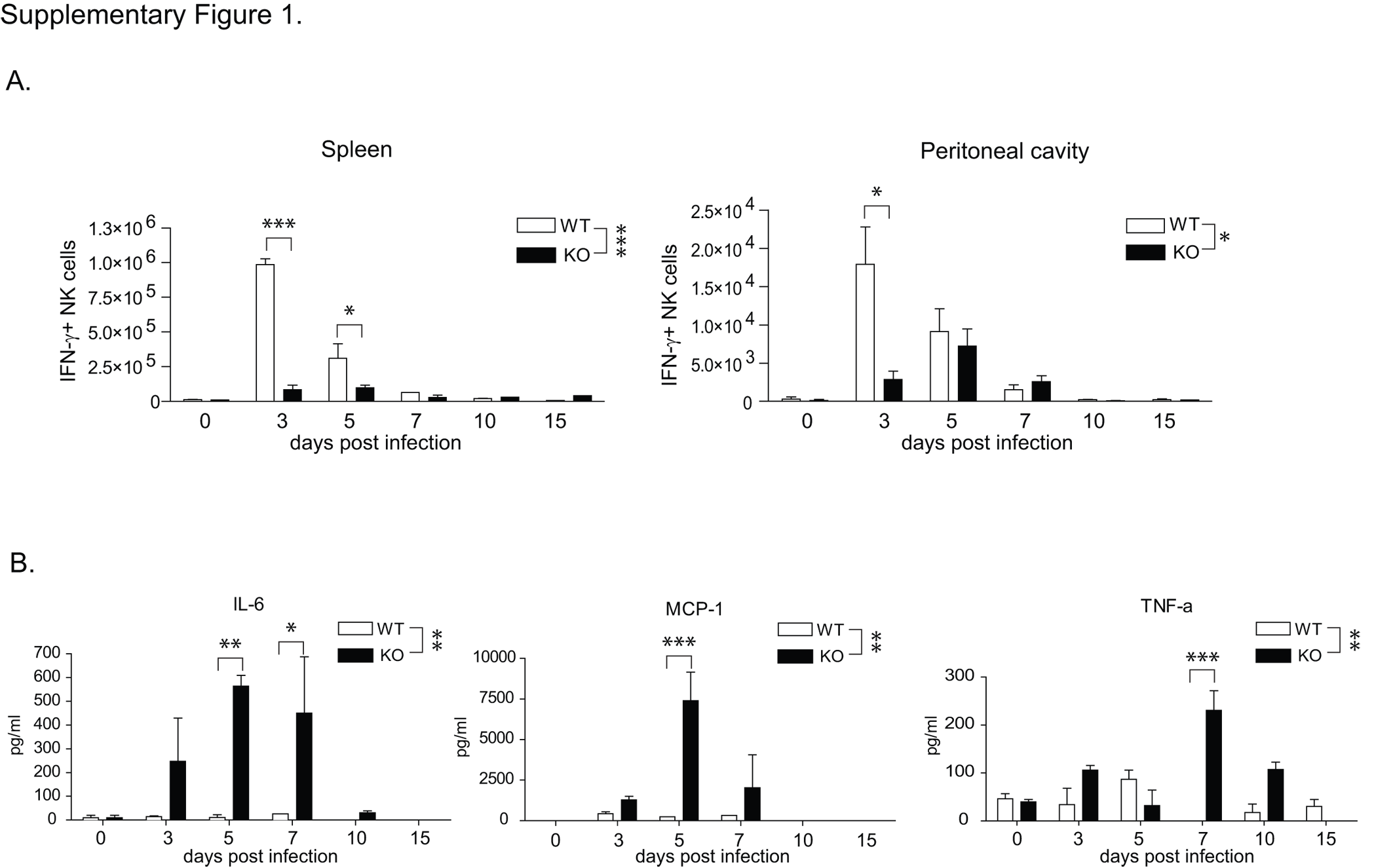

Supplement: FIG S1 [file mSphere.00634-20-sf001.tif]

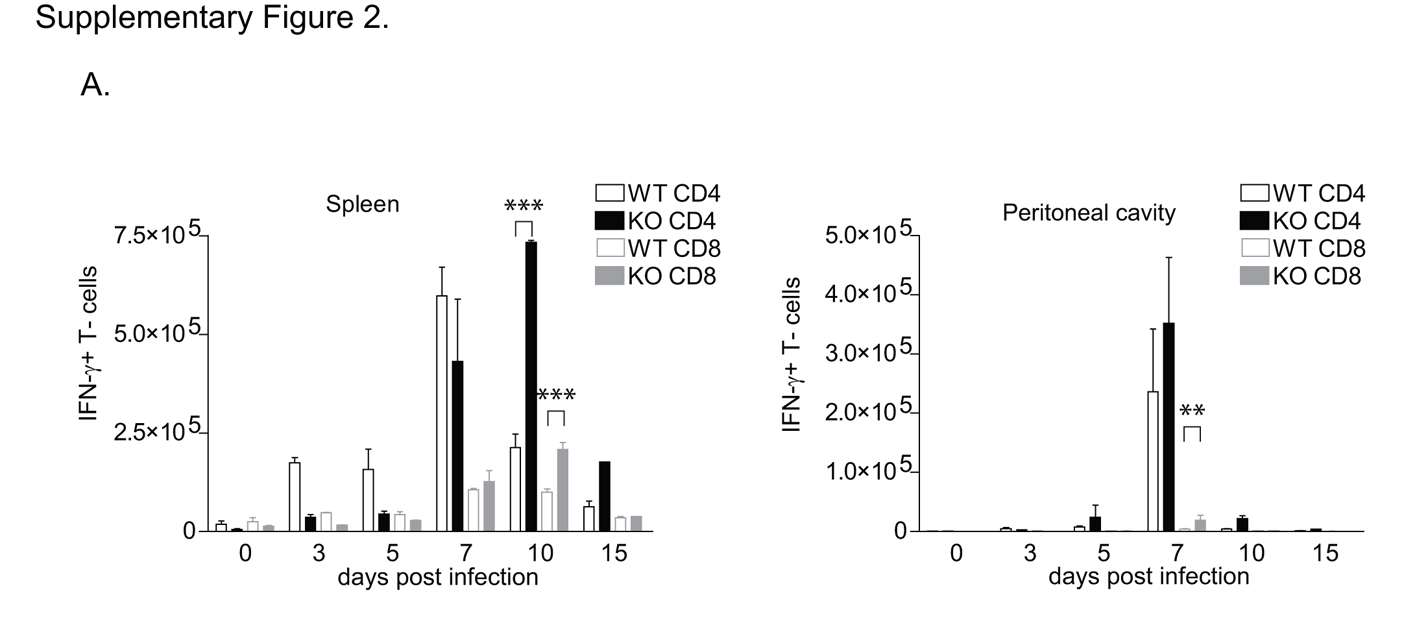

Supplement: FIG S2 [file mSphere.00634-20-sf002.tif]
